# Evaluation of the prognostic value of CD3, CD8, and FOXP3 mRNA expression in early‐stage breast cancer patients treated with anthracycline‐based adjuvant chemotherapy

**DOI:** 10.1002/cam4.1730

**Published:** 2018-09-21

**Authors:** Marinos Tsiatas, Konstantine T. Kalogeras, Kyriaki Manousou, Ralph M. Wirtz, Helen Gogas, Elke Veltrup, Flora Zagouri, Georgios Lazaridis, Angelos Koutras, Christos Christodoulou, George Pentheroudakis, Constantina Petraki, Dimitrios Bafaloukos, Dimitrios Pectasides, Paris Kosmidis, Epaminontas Samantas, Charisios Karanikiotis, Pavlos Papakostas, Meletios‐Athanassios Dimopoulos, George Fountzilas

**Affiliations:** ^1^ Department of Oncology Athens Medical Center Marousi Greece; ^2^ Laboratory of Molecular Oncology Hellenic Foundation for Cancer Research/Aristotle University of Thessaloniki Thessaloniki Greece; ^3^ Translational Research Section Hellenic Cooperative Oncology Group Athens Greece; ^4^ Section of Biostatistics Hellenic Cooperative Oncology Group Data Office Athens Greece; ^5^ STRATIFYER Molecular Pathology GmbH Cologne Germany; ^6^ First Department of Medicine Laiko General Hospital National and Kapodistrian University of Athens School of Medicine Athens Greece; ^7^ Department of Clinical Therapeutics Alexandra Hospital National and Kapodistrian University of Athens School of Medicine Athens Greece; ^8^ Department of Medical Oncology Faculty of Medicine School of Health Sciences Papageorgiou Hospital Aristotle University of Thessaloniki Thessaloniki Greece; ^9^ Division of Oncology Department of Medicine University Hospital University of Patras Medical School Patras Greece; ^10^ Second Department of Medical Oncology Metropolitan Hospital Piraeus Greece; ^11^ Department of Medical Oncology Ioannina University Hospital Ioannina Greece; ^12^ Department of Pathology Metropolitan Hospital Piraeus Greece; ^13^ First Department of Medical Oncology Metropolitan Hospital Piraeus Greece; ^14^ Oncology Section Second Department of Internal Medicine Hippokration Hospital Athens Greece; ^15^ Second Department of Medical Oncology Hygeia Hospital Athens Greece; ^16^ Third Department of Medical Oncology Agii Anargiri Cancer Hospital Athens Greece; ^17^ Department of Medical Oncology 424 Army General Hospital Thessaloniki Greece; ^18^ Oncology Unit Hippokration Hospital Athens Greece; ^19^ Aristotle University of Thessaloniki Thessaloniki Greece

**Keywords:** breast cancer, CD3, CD8, FOXP3, prognostic value, qRT‐PCR, receptor activator of nuclear factor‐kB, survival

## Abstract

**Background:**

Tumor‐infiltrating lymphocytes (TILs) have been shown to be of prognostic value in several cancer types. In early breast cancer, TILs have a prognostic utility, as well, especially in HER2‐positive and triple‐negative breast cancer. TILs presence is broadly associated with improved survival; however, there is controversy regarding TILs subpopulations.

**Patients and methods:**

Early‐stage breast cancer patients treated with anthracycline‐based chemotherapy within two randomized trials were included in the study. We evaluated, by qRT‐PCR, 826 tumor tissue samples for mRNA expression of CD3, CD8, and FOXP3 for potential prognostic significance in terms of disease‐free survival (DFS) and overall survival (OS).

**Results:**

After a median follow‐up of 133.0 months, 255 patients (30.9%) had died and 314 (38.0%) had disease progression. In the univariate analysis, high CD3 and CD8 mRNA expression was found to be of favorable prognostic value for DFS (*P* = 0.007 and *P* = 0.016, respectively). In multivariate analyses, the association of high CD8 mRNA expression with increased DFS was retained (HR = 0.77, 95% CI 0.60‐0.998, Wald's *P* = 0.048), whereas that of high CD3 mRNA expression was of marginal statistical significance (HR = 0.77, 95% CI 0.59‐1.01, *P* = 0.059). Moreover, a significant interaction was observed between HER2 status and CD3 mRNA expression with respect to DFS (interaction *P* = 0.032). In the HER2‐positive subgroup, the hazard ratio associated with high CD3 mRNA expression was of greater magnitude (HR = 0.48, 95% CI 0.30‐0.76, *P* = 0.002) compared with the hazard ratio presented above, for the entire cohort. No significant findings were observed for FOXP3 in terms of DFS, while none of the studied markers were of prognostic value for OS.

**Conclusions:**

High CD3 and CD8 mRNA expression in early‐stage breast cancer patients is of prognostic value for decreased risk of relapse and, in the future, could potentially be of importance in deciding the most appropriate therapeutic strategy in light of the recent immune‐related treatment developments.

## 1 INTRODUCTION

It has been well established that cancer evolution largely depends on the evasion of the immune system by the tumor cells. In this dynamic process, the immune system plays a dual role and may eliminate tumor cells, thus suppressing tumor progression, but on the other hand, it can promote their survival, facilitating tumor growth by specifically modifying the tumor microenvironment. Tumor‐infiltrating lymphocytes (TILs) have been extensively studied over the last two decades, and a growing body of evidence has provided insight on tumor immunogenicity.^1^, ^2^, ^3^ Initially, TILs were thought to be activated against tumor antigens and to be able to recognize and destroy cancer cells. Nevertheless, TILs are usually in an anergic state due to the ability of tumor cells to suppress antitumor immune activity, thus evading immunogenic death. This procedure, known as “immunoediting,” consists of three phases: the “elimination” phase, where immune effector cells recognize and eliminate cancer cells with great efficacy; the “equilibrium” phase, where tumor cells, following a selection procedure, gain a survival advantage and manage to evade recognition and elimination by the immune cells; and the late “escape” phase, where tumor is mainly characterized by an immunosuppressive environment, with the contribution of immune suppressor cells, leading to uncontrolled tumor growth.^4^

Breast cancer, unlike other tumor types, such as melanoma, was historically considered as nonimmunogenic. However, recent studies have demonstrated that breast tumors are infiltrated by lymphocytes, with high numbers of TILs being present in more aggressive subtypes, such as HER2‐positive and triple‐negative (TN). Overall, breast cancer patients with more than 50% or 60% lymphocyte infiltration of their tumor bed or stroma (lymphocyte predominant breast cancer, LPBC type) have improved clinical outcomes.^5^, ^6^ More specifically, in HER2‐positive and TN breast cancers, where the LPBC subtype is more common (approximately 20% of such tumors), even incremental increases in stromal or intratumoral TILs predict for better response to chemotherapy and survival.^5^, ^6^, ^7^, ^8^ TILs in these subtypes are predominantly T cells and CD8^+^ T cells represent the subpopulation that is closely associated with favorable outcomes in terms of survival, especially in TN breast cancer patients.^9^ In HER2‐positive breast cancers, the association of CD3^+^ and CD8^+^ T cells with improved survival is stronger in the hormone receptor‐negative than in the hormone receptor‐positive subtype, showing in part that hormone receptor positivity or negativity is a stronger factor than HER2 status in affecting T‐cell infiltration in breast cancer.^10^, ^11^

Indeed, a recent systematic review has shown that hormone receptor‐positive HER2‐negative breast cancers demonstrate the lowest percentage of T‐cell infiltration and the lowest incidence of LPBC (6% vs 20% in the TN subtype).^12^ This might be explained by the fact that luminal tumors have low expression of major histocompatibility complex molecules and, on the other hand, estrogen receptors are promoting a TH2 immune reaction, thus rendering these tumors less immunogenic, leading to decreased T‐cell infiltration.^13^, ^14^

Among TILs, there are subtypes that inhibit T‐cell activation, with FOXP3^+^ T‐regulatory (Treg) cells representing the major component of this inhibition. Tregs affect CD8^+^ function and inhibit dendritic cell‐mediated antigen presentation, as well as natural killer (NK) cell activation. However, the prognostic value of Tregs in breast cancer is controversial, as different studies have shown that Tregs are positively associated with poor survival,^15^ whereas other studies have demonstrated that high infiltration of Tregs is associated with better outcomes.^16^, ^17^

Moreover, the majority of breast cancer patients develop bone metastases, with the microenvironment of the host and its interactions with tumor cells playing a pivotal role in their progression.^18^ Receptor activator of nuclear factor‐kB (RANK) with its ligand (RANKL) and the RANKL decoy receptor osteoprotegerin (OPG) is highly involved in many cell functions associated with formation and progression of bone metastases; we have recently demonstrated that low RANKL mRNA expression in tumor tissue of early breast cancer patients is of prognostic significance for increased risk of relapse and bone metastases.^19^ RANK and RANKL are expressed not only in tumor tissue but also in immune cells. For example, RANK is expressed in tumor‐associated macrophages (TAMs) and NK cells, while RANKL has been reported to be expressed in activated T cells.^20^ Such expression may also play a role in the formation and progression of bone metastases, but this has yet to be elucidated.

In this study, we sought to investigate the prognostic value of T‐cell marker mRNA expression (CD3, CD8, and FOXP3), as well as their association with TILs and RANK, RANKL, and OPG mRNA expression, in a large cohort of early‐stage breast cancer patients treated with anthracycline‐based adjuvant chemotherapy in the context of two prospective phase III randomized trials.

## 2 PATIENTS AND METHODS

### 2.1 Patient population

This was a retrospective translational research study among 1681 patients with early breast cancer, enrolled in two prospective phase III adjuvant trials. The HE10/97 trial^21^ was a randomized phase III trial (ACTRN12611000506998) in patients with intermediate/high‐risk operable breast cancer, comparing four cycles of epirubicin (E) followed by four cycles of intensified CMF (E‐CMF) with three cycles of E, followed by three cycles of paclitaxel (T, Taxol^®^, Bristol Myers‐Squibb, Princeton, NJ) followed by three cycles of intensified CMF (E‐T‐CMF). The current definition of high‐risk breast cancer is based on the “International expert consensus on the primary therapy of early breast cancer 2007”.^22^ Specifically, high‐risk patients were node‐positive patients with 1‐3 involved lymph nodes and ER and PgR absent, or HER2/neu gene overexpressed or amplified; or node‐positive patients with four or more involved lymph nodes. The cycles were given every 2 weeks with G‐CSF support. Dose intensity of all drugs in both treatment arms was identical, but cumulative doses and duration of chemotherapy period differed. In total, 595 eligible patients entered the study in a period of 3.5 years (1997‐2000).

The HE10/00 trial^23^, ^24^ was a randomized phase III trial (ACTRN12609001036202), in which patients were treated with E‐T‐CMF (exactly as in the HE10/97 trial) or with four cycles of epirubicin/paclitaxel (ET) combination (given on the same day) every 3 weeks followed by three cycles of intensified CMF every 2 weeks (ET‐CMF). By study design, the cumulative doses and the chemotherapy duration were identical in the two arms but dose intensity of epirubicin and paclitaxel was double in the E‐T‐CMF arm. A total of 1086 eligible patients with node‐positive operable breast cancer were accrued in a period of 5 years (2000‐2005).

HER2‐positive patients received trastuzumab upon relapse, as previously described^25^; no anti‐HER2 treatment was given in the adjuvant setting. Treatment schedules for the two studies are shown in Table S1. Baseline characteristics and clinical outcomes of both trials have already been described.^21^, ^23^, ^24^, ^26^ Primary tumor diameter, axillary nodal status, and tumor grade were obtained from the pathology report. Clinical protocols were approved by local regulatory authorities, while the present translational research study was approved by the “Papageorgiou” Hospital Institutional Review Board (July 15, 2013) and the Bioethics Committee of the Aristotle University of Thessaloniki School of Medicine (December 18, 2013). All patients signed a study‐specific written informed consent before randomization, which in addition to giving consent for the trial allowed the use of biological material for future research purposes. All clinical investigations related to this study have been conducted according to the principles expressed in the Declaration of Helsinki.

### 2.2 Tissue microarray (TMA) construction

**Figure 1:**
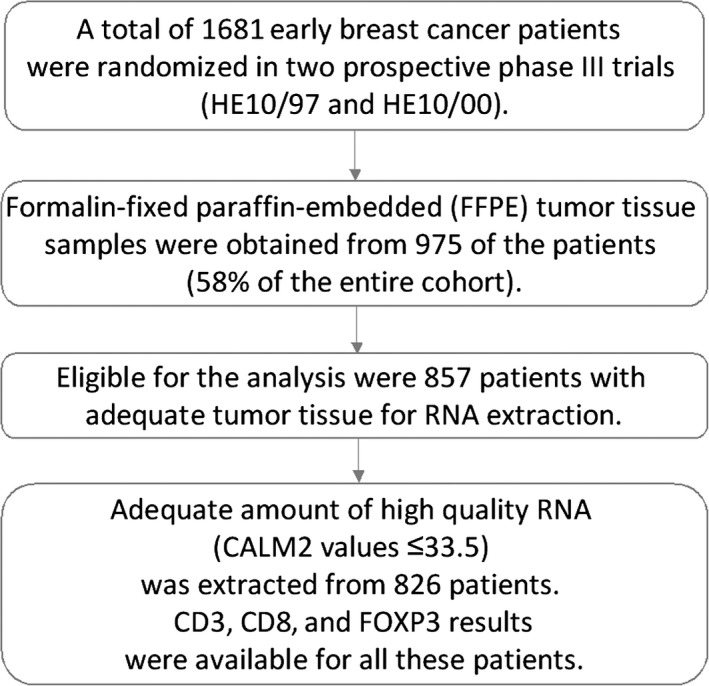
Consort diagram

Formalin‐fixed paraffin‐embedded (FFPE) tumor tissue samples from 975 patients (58.0% of 1681 randomized patients) were obtained during the initial breast surgery, before the initiation of adjuvant chemotherapy, and were collected retrospectively in the first trial (HE10/97) and prospectively in the second (HE10/00). The REMARK diagram^27^ for the study is shown in Figure 1. Hematoxylin‐eosin stained sections from the tissue blocks were reviewed by two experienced breast cancer pathologists, and the most representative tumor areas were marked for the construction of the ΤΜΑ blocks with the use of a manual arrayer (Model I, Beecher Instruments, San Prairie, WI), as previously described.^28^, ^29^ Each case was represented by two tissue cores, 1.5 mm in diameter, obtained from the most representative areas of primary invasive tumors or in some cases (9.6%) from synchronous axillary lymph node metastases, and re‐embedded in 51 microarray blocks. Each TMA block contained 38 to 66 tissue cores from the original tumor tissue blocks, while cores from various neoplastic, non‐neoplastic, and reactive tissues were also included, serving as orientation controls for slide‐based assays. Cases not represented, damaged, or inadequate on the TMA sections were recut from the original blocks, when material was available, and these sections were used for protein expression analysis. Stromal TILs density, that is, intratumoral stromal area occupied by mononuclear inflammatory cells over total intratumoral stromal area,^30^ was previously evaluated in whole H&E sections for the present cohort^6^; this variable was here assessed as continuous for associations and in 10% increments for outcome analyses.

### 2.3 Immunohistochemistry (IHC)

Immunohistochemical labeling was performed according to standard protocols on serial 2.5 μm thick sections from the original blocks or the TMA blocks. To assure optimal reactivity, immunostaining was applied 7 to 10 days after sectioning at the Laboratory of Molecular Oncology of the Hellenic Foundation for Cancer Research, Aristotle University of Thessaloniki School of Medicine. The staining procedures for HER2 (A0485 polyclonal antibody, dilution 1:200, Dako, Glostrup, Denmark), estrogen receptor (ER, clone 6F11, dilution 1:70, NovocastraTM, Leica Biosystems, Newcastle, UK), progesterone receptor (PgR, clone 1A6, dilution 1:70, NovocastraTM, Leica Biosystems), and Ki67 (clone MIB‐1, dilution 1:70, Dako) were performed using a Bond MaxTM autostainer (Leica Microsystems, Wetzlar, Germany), as previously described in detail.^31^, ^32^, ^33^, ^34^, ^35^

### 2.4 Interpretation of the IHC results

The evaluation of all IHC sections was made by two experienced breast cancer pathologists, blinded as to the patients' clinical characteristics and survival data, according to existing established criteria, as previously described.^25^ Briefly, HER2 protein expression was scored in a scale from 0 to 3+, the latter corresponding to uniform, intense membrane staining in >30% invasive tumor cells^36^; ER and PgR were considered positive if staining was present in ≥1% of tumor cell nuclei^37^; and, for Ki67, the expression was defined as low (<20%) or high (≥20%) based on the percentage of stained/unstained nuclei from the tumor areas.^38^ If one of the tissue cores was lost or damaged, the overall score was determined from the remaining one. When whole tissue sections were used, the entire tumor area was evaluated.

### 2.5 Fluorescence in situ hybridization (FISH)

TMA sections or whole tissue sections (5 μm thick) were used for FISH analysis, using the ZytoLight^®^ SPEC HER2/TOP2A/CEP17 triple color probe (Z‐2073, ZytoVision, Bremerhaven, Germany), as previously described.^39^ FISH was performed according to the manufacturer's protocol with minor modifications in all cases, not only the HER2 IHC 2+ cases.

Digital images were constructed using specifically developed software for cytogenetics (XCyto‐Gen, ALPHELYS, Plaisir, France). Processed sections were considered eligible for FISH evaluation according to the ASCO/CAP criteria.^36^ For the evaluation of the HER2 gene status, nonoverlapping nuclei from the invasive part of the tumor were randomly selected, according to morphological criteria using DAPI staining, and scored. Twenty tumor nuclei were counted according to Press et al.^40^ The HER2 gene was considered to be amplified when the HER2/CEP17 ratio was >2.2,^36^ or the mean HER2 copy number was >6.^41^ In cases with values at or near the cutoff (1.8‐2.2), 20‐40 additional nuclei were counted and the ratio was recalculated. In cases with a borderline ratio, additional FISH assays were performed in whole sections.^42^ The data from the evaluation of TOP2A gene status were neither analyzed nor presented in the present manuscript.

### 2.6 RNA isolation and quantitative reverse transcription‐polymerase chain reaction (qRT‐PCR) assessment

Prior to RNA isolation, macrodissection of tumor areas was performed in most (69%) of the FFPE sections (all sections with <50% tumor cell content). More than one FFPE section (2‐8 sections, 10 μm thick) was used for RNA extraction when the tumor surface of a given sample was less than 0.25 cm^2^. From each FFPE section or macrodissected tissue fragments, RNA was extracted using a standardized fully automated isolation method for total RNA from FFPE tissue, based on germanium‐coated magnetic beads (XTRAKT kit, STRATIFYER Molecular Pathology GmbH, Cologne, Germany) in combination with a liquid handling robot (XTRAKT XL, STRATIFYER Molecular Pathology GmbH), as previously described in detail.^19^, ^32^, ^34^, ^35^, ^43^, ^44^ The method involves extraction‐integrated deparaffinization and DNase I digestion steps. The quality and quantity of RNA were checked by measuring CALM2 expression as a surrogate for amplifiable mRNA by qRT‐PCR. CALM2 was used as endogenous reference, as it had previously been identified as being highly and stably expressed among breast cancer tissue samples.^45^ Of the 975 FFPE tumor tissue samples collected, 857 (87.9%) had enough material left for RNA isolation needed for this study.

qRT‐PCR primers and labeled hydrolysis probes were selected using Primer Express^®^ Software, versions 2.2 and 3 (Applied Biosystems/Life Technologies, Karlsruhe, Germany), according to the manufacturer's instructions, and were controlled for single nucleotide polymorphisms. All primers, probes, and amplicons were checked for their specificity against nucleotide databases at NCBI using Basic Local Alignment Search Tool (BLAST). Primers and probes were purchased from Eurogentec S.A. (Seraing, Belgium). For each primer/probe set, the amplification efficiency was tested, aiming to reach comparable efficiency of >90% (efficiency range from 91% to 108%). Primers and hydrolysis probes were diluted to 100 μmol/L, using a stock solution with nuclease‐free water (Life Technologies GmbH, Darmstadt, Germany).^19^, ^34^, ^35^, ^44^ qRT‐PCR was applied for the relative quantification (RQ) of RANK, OPG, and RANKL. The Primer/Probe (YakimaYellow/FAM‐labeled) sets used for amplification of the target and reference genes are shown in Table 1.

**Table 1:** Primer and probe sequences used for quantitative reverse transcription‐polymerase chain reaction (qRT‐PCR)

Gene symbol	NM_Number	Probe name	Probe sequence	Forward name	Forward sequence	Reverse name	Reverse sequence
CD3	NM_198053	MP317	CAAAGCTCTGTGCCTCTGTAATCGGCA	MP317_For	CACCGCGGCCATCCT	MP317_Rev	AGTTTGGGATCCAGCAGGC
CD8	NM_001145873	MP607	TTCCTGCCAGCGAAGCCCAC	MP607_For	TGAGCAACTCCATCATGTACTTCAG	MP607_Rev	GGCGCCGGTGTTGGT
FOXP3	NM_014009	MP551	TTTTCTGTCAGTCCACTTCACCAAGCCTG	MP551_For	CCCACAAGCCAGGCTGAT	MP551_Rev	GCATCGGGTCCTTGTCCA
CALM2	NM_001743	MP501	TCGCGTCTCGGAAACCGGTAGC	MP501_For	GAGCGAGCTGAGTGGTTGTG	MP501_Rev	AGTCAGTTGGTCAGCCATGCT

For PCR, 0.5 μmol/L of each primer and 0.25 μmol/L of each probe were used. All quantitative reverse transcription PCRs were performed in duplicates using the SuperScript^®^ III Platinum^®^ One‐Step qRT‐PCR kit (Invitrogen/Life Technologies, Darmstadt, Germany) according to the manufacturer's instructions. Experiments were performed on a Stratagene Mx3005p (Agilent Technologies, Waldbronn, Germany) with 30 minutes at 50°C and 2 minute at 95°C followed by 40 cycles of 15  seconds at 95°C and 30 seconds at 60°C. The lengths of the amplicons detected by the CD3, CD8, FOXP3, and CALM2 assays were 72, 97, 71, and 72 bp, respectively, with PCR efficiencies [*E* = 1(10‐slope)] of 106%, 91%, 103%, and 99%, respectively. Samples were considered eligible for further investigation (N = 826, Figure 1) when the cycle threshold (CT) values of the housekeeping gene were ≤33.5 (duplicate mean values). When the difference between the duplicate CT values for a given sample was >0.50, the sample was reassessed in triplicates (repeats). Relative expression levels of the target transcripts were calculated as 40‐DCT values (DCT = mean CT target gene − mean CT housekeeping gene) to yield positively correlated numbers and to facilitate comparisons.^34^, ^35^, ^44^ CD3, CD8, and FOXP3 results were available for all 826 eligible samples, after the above‐mentioned repeats were completed. A commercially available human reference RNA (Stratagene qPCR Human Reference Total RNA, Agilent Technologies) was used as positive control. No‐template controls were assessed in parallel to exclude contamination. The qRT‐PCR method has recently been validated.^45^

### 2.7 Statistical analysis

A total of 826 patients with breast cancer were included in this study [Figure 1]. Continuous variables are presented as means (standard deviation) and medians (range), while categorical variables are presented as frequencies (percent, %). The chi‐square test was used for group comparisons of categorical data, while Kruskal–Wallis or Mann–Whitney *U* tests were used for the comparison of continuous variables between groups, as appropriate. Spearman's correlation coefficient was used for estimating the correlations between continuous variables.

Overall survival (OS) was defined as the time (in months) from the date of diagnosis with breast cancer to the date of patient's death or last contact, while disease‐free survival (DFS) was defined as the time (in months) from the date of diagnosis to documented first relapse, death without prior documented relapse or last contact, whichever occurred first.^46^ Surviving patients (for OS and DFS) and patients without relapse (for DFS) were censored at the date of last contact. Women who died without prior relapse were treated as having had relapse at the date of their death. Survival curves were estimated using the Kaplan–Meier method and compared across groups with the log‐rank test. The associations between the factors examined and mortality/relapse rate were evaluated with hazard ratios estimated with Cox proportional hazards model. The proportional hazards assumption was tested by evaluating the statistical significance of the time‐dependent associations between each variable and relapse/death rates.

The following parameters were studied in relation to DFS/OS: (a) clinicopathological, such as age (≤median, >median), positive lymph nodes (0‐3, ≥4 positive lymph nodes), tumor size (≤2, 2‐5, >5 cm), chemotherapy treatment with paclitaxel (no, yes), adjuvant hormonal therapy (no, yes), adjuvant radiotherapy (no, yes), breast surgery (breast‐conserving surgery, modified radical mastectomy), subtypes (luminal A, luminal B, luminal‐HER2, HER2‐enriched, triple‐negative), Ki67 (continuous), and TILs (10% increments), (b) T‐cell mRNA markers considered as 2‐level categorical variables (high expression vs low expression) using the 50th percentile (median value) as a cutoff: CD3, CD8, and FOXP3.

We also assessed whether the association of the T‐cell mRNA markers was modified by treatment or breast cancer subtype by adding interaction terms in Cox regression analyses between CD3, CD8, and FOXP3 and: chemotherapy treatment with paclitaxel (yes vs no); HER2 status; and ER/PgR status. In multivariate analyses, we estimated the effect (HR) of each of the T‐cell mRNA markers adjusted for the effect of the clinicopathological parameters that were statistically significant or marginally significant in the univariate analysis (*P* < 0.10).

Finally, the associations between the three T‐cell mRNA markers (CD3, CD8, and FOXP3) and the following mRNA markers were examined: RANK (median cutoff: high, low), RANKL (median cutoff: high, low), and OPG (median cutoff: high, low). The bivariate correlations between all mRNA markers, assessed in their original form as continuous variables, were also estimated.

Results of this study were presented according to reporting recommendations for tumor marker prognostic studies.^27^ This study is prospective–retrospective as described in Simon et al^47^ All analyses were performed in the entire cohort. The statistical analyses were performed using the SAS software (SAS for Windows, version 9.4, SAS Institute Inc., Cary, NC). Statistical significance was set at 2‐sided *P* = 0.05.

## 3 RESULTS

### 3.1 Patient characteristics

Selected patient and tumor characteristics of the 826 patients that were included in the analysis are presented in Table 2. Median age at diagnosis was 52.7 years (range: 22‐79), while the majority of patients were postmenopausal (54.2%), ER/PgR‐positive (79.0%), and HER2‐negative (76.3%).

**Table 2:** Selected patient and tumor characteristics (N = 826)

Parameters	N (%)
Age (y)	
N	826
Mean (SD)	53.0 (11.3)
Median (min‐max)	52.7 (22‐79)
Age (median cutoff)	
≤52.7	415 (50.2%)
>52.7	411 (49.8%)
Menopausal status	
Postmenopausal	448 (54.2%)
Premenopausal	378 (45.8%)
Breast surgery	
Breast‐conserving surgery	242 (29.3%)
Modified radical mastectomy	583 (70.6%)
Not reported	1 (0.1%)
Tumor size (cm)	
≤2	252 (30.5%)
2‐5	472 (57.1%)
>5	102 (12.3%)
Positive lymph nodes	
0‐3	335 (40.6%)
≥4	491 (59.4%)
Treatment group	
E‐CMF	141 (17.1%)
E‐T‐CMF	396 (47.9%)
ET‐CMF	289 (35.0%)
Adjuvant hormonal therapy	
No	150 (18.2%)
Yes	660 (79.9%)
Not reported	16 (1.9%)
Adjuvant radiotherapy	
No	181 (21.9%)
Yes	624 (75.5%)
Not reported	21 (2.5%)
Histological grade	
I‐II	409 (49.5%)
III‐IV	416 (50.4%)
Not reported	1 (0.1%)
TNM stage	
IA	4 (0.5%)
IIA	113 (13.7%)
IIB	189 (22.9%)
IIIA	340 (41.2%)
IIIC	180 (21.8%)
ER/PgR status	
Informative N (%)	770 (93.2%)
Positive	608 (79.0%)
Negative	162 (21.0%)
HER2 status	
Informative N (%)	778 (94.2%)
Positive	184 (23.7%)
Negative	594 (76.3%)
Ki67	
Informative N (%)	767 (92.9%)
Mean (SD)	31.4 (24.3)
Median (min‐max)	25 (0‐98)
TILs	
Informative N (%)	786 (95.2%)
Mean (SD)	12.6 (14.8)
Median (min‐max)	8 (0‐90)
Subtypes^a^	
Informative N (%)	757 (91.6%)
Luminal A	229 (30.3%)
Luminal B	263 (34.7%)
Luminal‐HER2	103 (13.6%)
HER2‐enriched	78 (10.3%)
Triple‐negative	84 (11.1%)

a Breast cancer subtypes defined by immunohistochemistry: luminal A (ER*‐positive* and/or PgR‐positive, *HER2‐negative, Ki67* ^*low*^); luminal B (ER*‐positive* and/or PgR‐positive, *HER2‐negative, Ki67* ^*high*^); luminal‐HER2 (ER*‐positive* and/or PgR‐positive, HER2‐positive); HER2‐enriched (ER‐negative, PgR‐negative, HER2‐positive); and triple‐negative (ER‐negative, PgR‐negative, and HER2‐negative).

**Figure 2:**
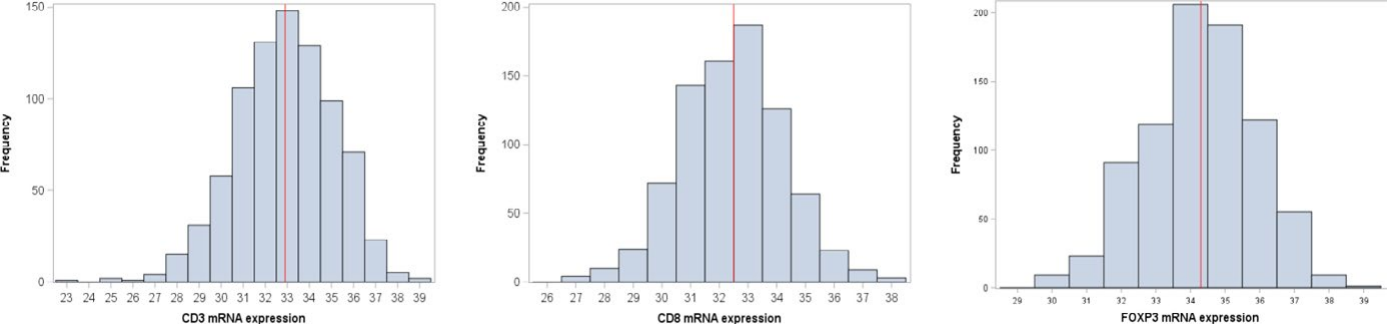
Histograms of CD3, CD8, and FOXP3 mRNA expression (40‐DCT values). Red line represents the 50th percentile (median)

**Figure 3:**
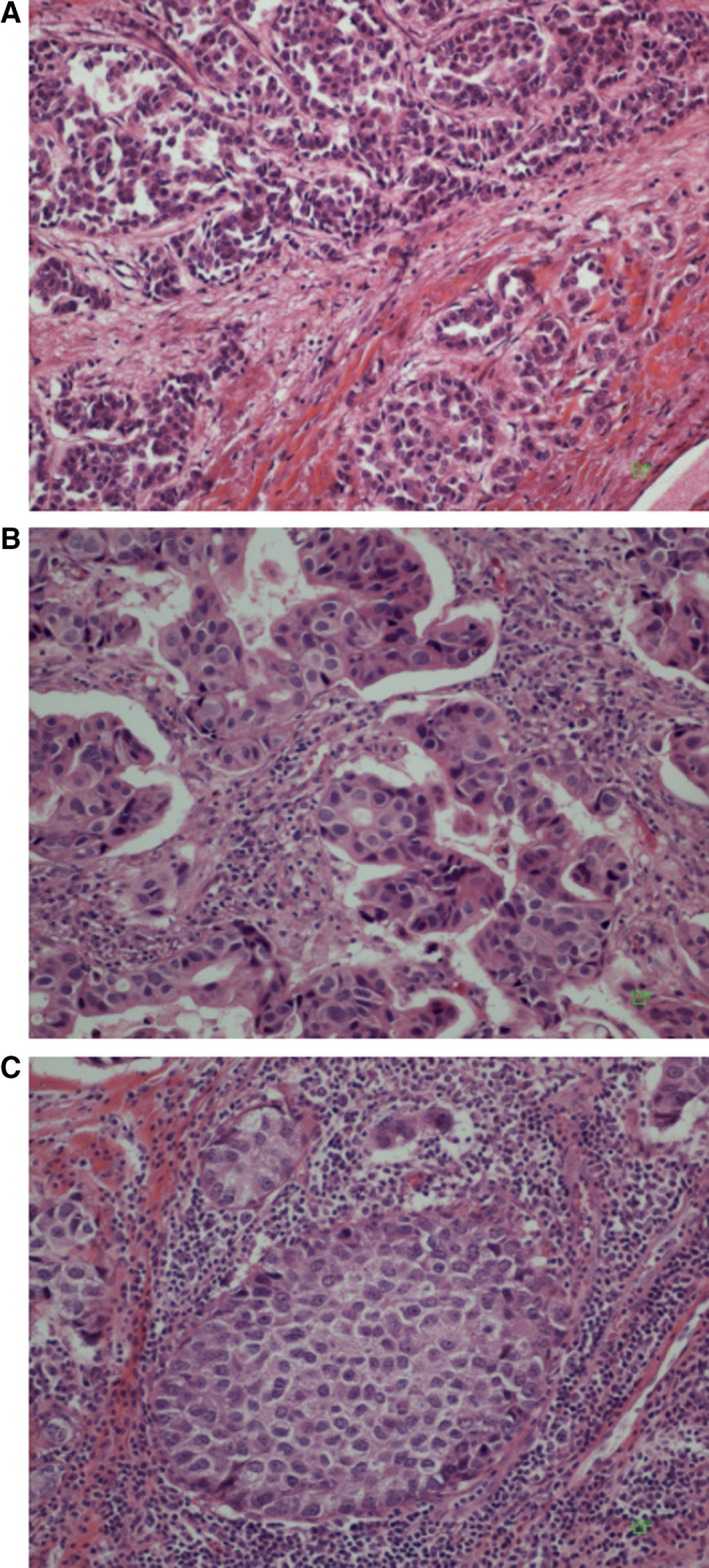
Representative pictures showing different stromal tumor‐infiltrating lymphocyte (TIL) densities in the examined breast tumors. All tumors are ductal carcinomas of the nonspecific type. Original magnification ×200. A, grade II, 5% TIL density; B, grade III, partially mucous secreting, 40% TIL density; C, grade III, 80% TIL density

The distribution of tumor samples based on the normalized expression of mRNA encoding for the three examined markers is presented in Figure 2. The median value of CD3, CD8, and FOXP3 mRNA expressions was 32.9 (range: 23.4‐38.6), 32.5 (26.8‐38.4), and 34.3 (29.5‐38.9), respectively. Representative pictures showing different stromal tumor‐infiltrating lymphocyte (TIL) densities in the examined breast tumors are shown in Figure 3.

### 3.2 Association of mRNA markers with clinicopathological characteristics

**Figure 4:**
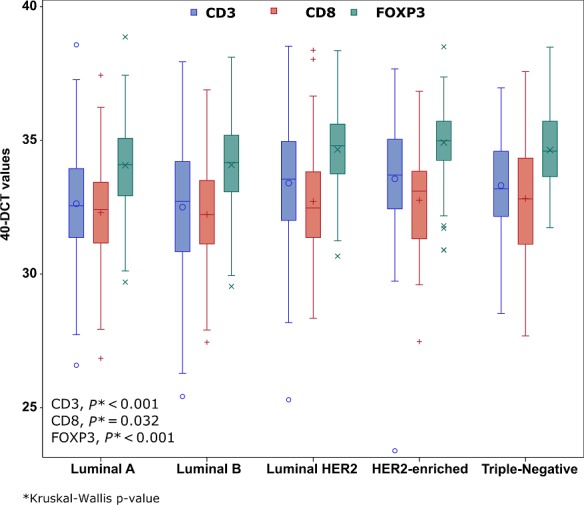
Distribution of the study markers by breast cancer subtypes

The associations of the T‐cell mRNA markers and selected clinicopathological parameters are presented in Table 3. ER/PgR‐negative, HER2‐positive, and grade III‐IV tumors had higher CD3 (all Mann–Whitney *U P*‐values ≤0.001), CD8 (*P* = 0.007, *P* = 0.038, and *P* = 0.002, respectively) and FOXP3 (all *P*‐values <0.001) mRNA expression. In addition, tumors of lower size were found to have higher mRNA expression of CD3 (Kruskal–Wallis test, *P* = 0.001), CD8 (*P* < 0.001), and FOXP3 (*P* = 0.018), while lower number of positive lymph nodes was associated with higher FOXP3 mRNA expression (Mann–Whitney *U* test, *P* = 0.004). Postmenopausal women were found to have higher mRNA expression of FOXP3 (*P* < 0.001) and so did older women (age above the median). Finally, CD3, CD8, and FOXP3 mRNA expressions were significantly associated with breast cancer subtypes (Kruskal–Wallis test, *P* < 0.001, *P* = 0.032 and *P* < 0.001, respectively). The distribution of the study markers by breast cancer subtypes is presented in Figure 4.

**Table 3:** Associations between continuous mRNA expression values and selected clinicopathological characteristics

	CD3				CD8				FOXP3			
	N	Median	Range	*P*‐value	N	Median	Range	*P*‐value	N	Median	Range	*P*‐value
Age (median cutoff)												
≤52.7	415	32.8	(23.4‐37.9)	0.16	415	32.5	(26.8‐38.4)	0.35	415	34.2	(29.5‐38.5)	**<0.001** a
>52.7	411	33.0	(25.4‐38.6)		411	32.5	(27.4‐38.0)		411	34.6	(29.7‐38.9)	
Menopausal status												
Postmenopausal	448	33.0	(25.4‐38.6)	0.66	448	32.6	(27.4‐38.0)	0.19	448	34.6	(29.7‐38.9)	**<0.001** a
Premenopausal	378	32.9	(23.4‐37.9)		378	32.4	(26.8‐38.4)		378	34.1	(29.5‐38.5)	
Breast surgery												
Breast‐conserving	242	33.4	(23.4‐38.6)	**0.014** a	242	32.6	(27.5‐37.4)	0.18	242	34.6	(30.1‐38.9)	**0.013** a
Modified radical mastectomy	583	32.8	(25.3‐38.5)		583	32.5	(26.8‐38.4)		583	34.2	(29.5‐38.5)	
Tumor size (in cm)												
≤2	252	33.2	(27.2‐38.6)	**0.001** c	252	32.8	(26.8‐37.6)	**<0.001** c	252	34.5	(30.0‐38.9)	**0.018** c
2‐5	472	32.8	(23.4‐38.5)		472	32.4	(27.2‐38.4)		472	34.4	(29.5‐38.4)	
>5	102	32.7	(26.6‐37.9)		102	32.0	(28.2‐36.0)		102	33.8	(30.1‐37.8)	
Positive lymph nodes												
0‐3	335	33.1	(25.3‐38.6)	0.10	335	32.6	(26.8‐38.0)	0.64	335	34.5	(29.5‐38.9)	**0.004** a
≥4	491	32.8	(23.4‐37.9)		491	32.4	(27.2‐38.4)		491	34.2	(30.0‐38.5)	
Paclitaxel treatment												
Non paclitaxel‐treated	141	32.1	(23.4‐36.0)	**<0.001** a	141	31.8	(26.8‐36.3)	**<0.001** a	141	33.6	(29.5‐36.9)	**<0.001** a
Paclitaxel‐treated	685	33.1	(25.3‐38.6)		685	32.6	(27.7‐38.4)		685	34.5	(29.7‐38.9)	
Adjuvant hormonal therapy												
No	150	33.9	(28.5‐37.9)	**<0.001** a	150	33.2	(28.9‐36.7)	**<0.001** a	150	35	(30.1‐38.5)	**<0.001** a
Yes	660	32.8	(23.4‐38.6)		660	32.3	(26.8‐38.4)		660	34.2	(29.5‐38.9)	
Adjuvant radiotherapy												
No	181	33.1	(28.0‐38.5)	0.52	181	32.6	(26.8‐38.0)	0.85	181	34.3	(29.5‐38.4)	0.26
Yes	624	32.9	(23.4‐38.6)		624	32.5	(27.2‐38.4)		624	34.3	(29.9‐38.9)	
Histological grade												
I‐II	409	32.7	(23.4‐38.6)	**0.001** a	409	32.2	(27.2‐37.4)	**0.002** a	409	34.0	(29.5‐38.9)	**<0.001** a
III‐IV	416	33.2	(25.3‐38.5)		416	32.7	(26.8‐38.4)		416	34.7	(29.9‐38.5)	
ER/PgR status												
Negative	162	33.5	(23.4‐37.7)	**<0.001** a	162	32.9	(27.5‐37.6)	**0.007** a	162	34.8	(30.9‐38.5)	**<0.001** a
Positive	608	32.8	(25.3‐38.6)		608	32.3	(26.8‐38.4)		608	34.2	(29.5‐38.9)	
HER2 status												
Negative	594	32.7	(25.4‐38.6)	**<0.001** a	594	32.4	(26.8‐37.6)	**0.038** a	594	34.2	(29.5‐38.9)	**<0.001** a
Positive	184	33.6	(23.4‐38.5)		184	32.7	(27.5‐38.4)		184	34.9	(30.7‐38.5)	
Ki67												
Low (<20%)	295	32.6	(26.6‐38.6)	**0.038** a	295	32.5	(26.8‐37.4)	0.59	295	34.2	(29.7‐38.7)	0.15
High (≥20%)	472	33.1	(23.4‐38.5)		472	32.5	(27.4‐38.4)		472	34.4	(29.5‐38.5)	
Subtypes^b^												
Luminal A	229	32.6	(26.6‐38.6)	**<0.001** c	229	32.4	(26.8‐37.4)	**0.032** c	229	34.1	(29.7‐38.9)	**<0.001** c
Luminal B	263	32.7	(25.4‐37.9)		263	32.2	(27.4‐36.9)		263	34.2	(29.5‐38.1)	
Luminal‐HER2	103	33.5	(25.3‐38.5)		103	32.5	(28.3‐38.4)		103	34.8	(30.7‐38.4)	
HER2‐enriched	78	33.7	(23.4‐37.7)		78	33.1	(27.5‐36.8)		78	35.0	(30.9‐38.5)	
Triple‐negative	84	33.2	(28.5‐37.0)		84	32.8	(27.7‐37.6)		84	34.6	(31.7‐38.5)	

Significant *P*‐values are shown in bold.

a Mann–Whitney *U* test.

b For breast cancer subtype classification please see footnote to Table 2.

c Kruskal–Wallis test.

### 3.3 Association among mRNA markers

**Figure 5:**
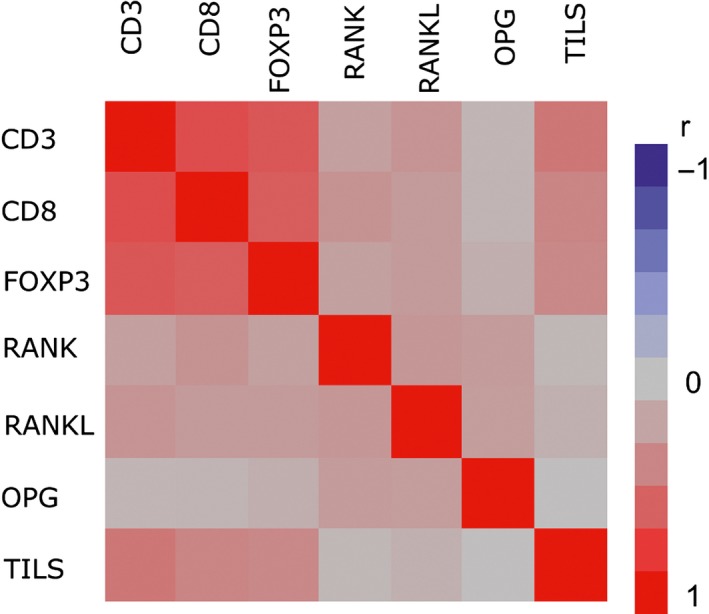
Heatmap of the Spearman correlations between all study markers

CD3 mRNA expression was significantly associated with CD8, FOXP3, RANK, and RANKL mRNA expressions (chi‐square test, all *P*‐values <0.001) (Table S2). More specifically, high expression of CD3 (≥median) was associated with high expression of the rest mRNA markers. Similar were the results for the CD8 and FOXP3 markers, which were also positively associated with one another, as well as with RANK and RANKL mRNA markers. In addition, FOXP3 was positively associated with OPG mRNA expression (*P* = 0.041). Using the continuous measurements of the mRNA markers, the strongest and statistically significant (*P* < 0.001) correlations were observed among the T‐cell markers: CD3 and CD8 (*r* = 0.70); CD3 and FOXP3 (*r* = 0.65); and CD8 and FOXP3 (*r* = 0.61) (Table S3). Finally, mRNA expression in all three markers was positively correlated with TILs, *r* = 0.52 for CD3, *r* = 0.41 for CD8 and *r* = 0.47 for FOXP3 (all *P*‐values <0.001) (Table S3). Heatmap of the Spearman correlations between all study markers is shown in Figure 5.

### 3.4 Lymph node mRNA expression

CD3, CD8, and FOXP3 mRNA expressions were also measured in 90 available lymph node samples that were paired to the primary tumor samples. A significant correlation of small to medium magnitude was observed between primary tumor and lymph node mRNA expression for the CD8 and FOXP3 markers; Spearman's *r* = 0.27 (*P* = 0.010) and *r* = 0.29 (*P* = 0.005), respectively. No significant correlation in the CD3 expression between the two tissues was observed (*r* = 0.16, *P* = 0.12) [results are not presented].

### 3.5 Markers effect on outcome

Survival status of all patients was updated in June 2014. Within a median follow‐up time of 133 months (range 0.1‐218.8), 255 deaths (30.9%) and 314 relapses occurred (38%). Of note, 279 of the 314 relapsed patients (88.9%) had bone metastases. Median OS was reached at 201.1 months, while median DFS was 201.3 months.

**Figure 6:**
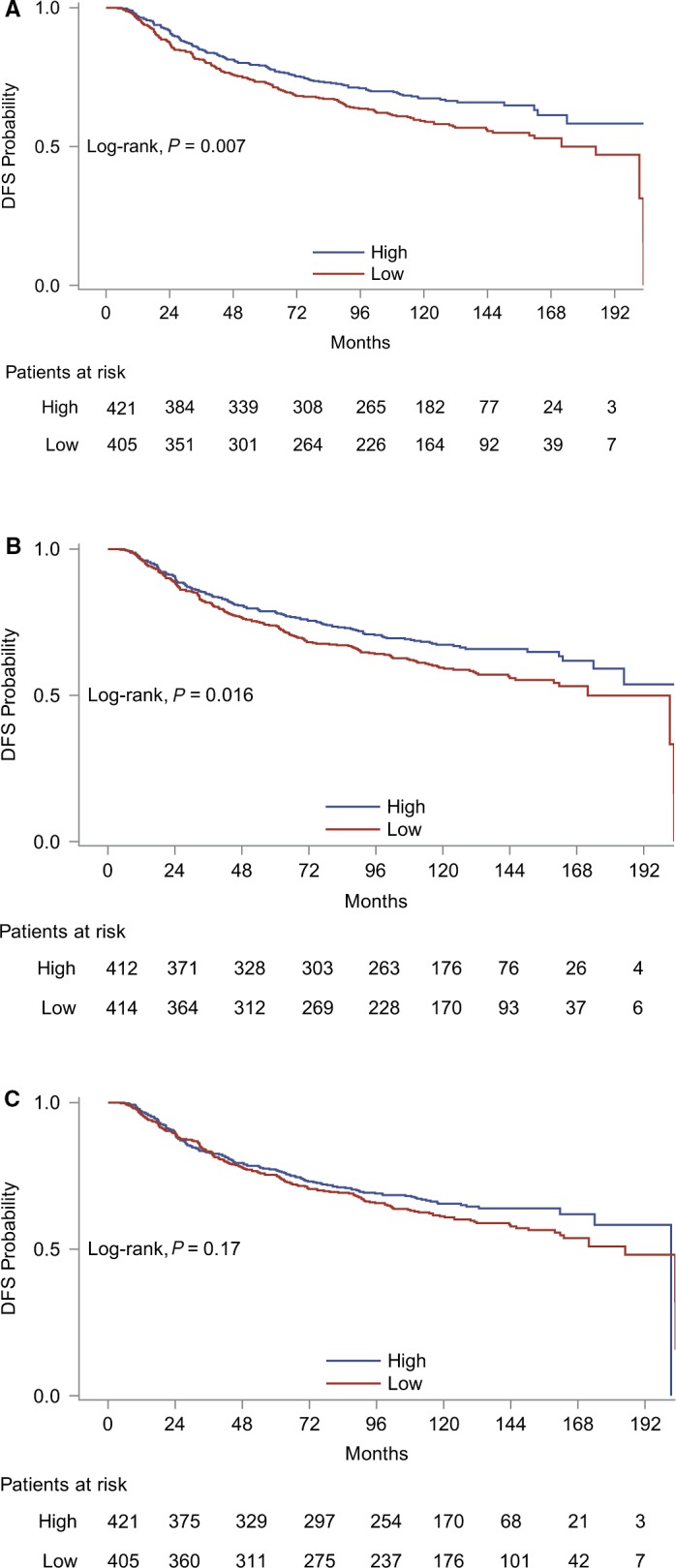
Kaplan–Meier curves for CD3 (A), CD8 (B), and FOXP3 (C) mRNA expression (using the median as a cutoff point) with respect to DFS

Results from univariate Cox regression analyses for each of the three T‐cell markers with respect to DFS and OS are presented in Table S4. Low, as compared to high, CD3 and CD8 mRNA expression was associated with increased relapse rate. More specifically, patients with low CD3 and CD8 mRNA expression had, respectively, 36% and 32% increased risk of relapse compared to patients with high mRNA expression of those markers. Kaplan–Meier curves for DFS based on CD3, CD8, and FOXP3 mRNA expressions are presented in Figure 6. FOXP3 mRNA expression was not significantly associated with DFS. Finally, none of the markers had a significant effect on mortality rate.

Results from univariate Cox regression analyses in the entire cohort for each of the clinicopathological parameters are presented in Table S5. Age, breast surgery, menopausal status, tumor size, positive lymph nodes, ER/PgR status, Ki67, TILs, subtypes, adjuvant hormonal therapy, and adjuvant radiotherapy were statistically significant or marginally significant in the univariate analysis for DFS and OS. Results from multivariate analyses including the aforementioned clinicopathological parameters (excluding menopausal status and ER/PgR status/hormonal therapy/Ki67 due to their high correlation with age and subtypes, respectively) and each of the significant T‐cell mRNA markers with respect to DFS are presented in Table 4. The HRs associated with CD3 and CD8 mRNA expression adjusted for the rest of the clinicopathological variables were of similar magnitude as the unadjusted hazard ratios. More specifically, low CD3, as well as low CD8 mRNA expression, remained unfavorable prognostic factors for DFS (adjusted HR = 1.30, 95% CI 0.99‐1.69, *P* = 0.059 and HR = 1.30, 95% CI 1.00‐1.68, *P* = 0.048, respectively).

**Table 4:** Hazard ratios and 95% CIs estimated from multivariate Cox regression with respect to DFS for each of the study markers that were significant in the univariate analyses (N = 705)

Parameters	Categories	N patients	N events	HR	95% CI		Wald's *P*
Model 1							
CD3 (median cut‐off)	Low vs High	351 vs 354	153 vs 112	1.30	0.99	1.69	**0.059**
Age	>52.7 vs ≤52.7	352 vs 353	146 vs 119	1.23	0.96	1.58	0.098
Breast surgery	MRM vs Breast‐conserving	494 vs 211	205 vs 60	1.40	1.03	1.90	**0.033**
Tumor size	2‐5 vs ≤2	406 vs 212	157 vs 65	1.19	0.88	1.59	0.26
	>5 vs ≤2	87 vs 12	43 vs 65	1.39	0.93	2.06	0.11
Positive lymph nodes	≥4 vs 0‐3	426 vs 279	201 vs 64	2.09	1.50	2.93	**<0.001**
Subtypes^a^	Luminal B vs Luminal A	242 vs 210	93 vs 66	1.27	0.92	1.74	0.15
	Luminal‐HER2 vs Luminal A	95 vs 210	39 vs 66	1.69	1.12	2.55	**0.013**
	HER2‐enriched vs Luminal A	76 vs 210	28 vs 66	1.44	0.91	2.27	0.12
	Triple‐negative vs Luminal A	82 vs 210	39 vs 66	2.15	1.43	3.23	**<0.001**
Adjuvant radiotherapy	Yes vs No	550 vs 155	220 vs 45	1.02	0.69	1.50	0.92
TILs (10% increments)				0.89	0.81	0.99	**0.031**
Model 2							
CD8 (median cutoff)	Low vs High	351 vs 354	153 vs 112	1.30	1.00	1.68	**0.048**
Age	>52.7 vs ≤52.7	352 vs 353	146 vs 119	1.22	0.95	1.56	0.12
Breast surgery	MRM vs Breast‐conserving	494 vs 211	205 vs 60	1.41	1.03	1.91	**0.030**
Tumor size	2‐5 vs ≤2	406 vs 212	157 vs 65	1.17	0.88	1.57	0.29
	>5 vs ≤2	87 vs 12	43 vs 65	1.35	0.90	2.01	0.14
Positive lymph nodes	≥4 vs 0‐3	426 vs 279	201 vs 64	2.12	1.52	2.97	**<0.001**
Subtypes^a^	Luminal B vs Luminal A	242 vs 210	93 vs 66	1.25	0.91	1.71	0.18
	Luminal‐HER2 vs Luminal A	95 vs 210	39 vs 66	1.63	1.08	2.46	**0.020**
	HER2‐enriched vs Luminal A	76 vs 210	28 vs 66	1.40	0.89	2.20	0.15
	Triple‐negative vs Luminal A	82 vs 210	39 vs 66	2.13	1.42	3.20	**<0.001**
Adjuvant radiotherapy	Yes vs No	550 vs 155	220 vs 45	1.02	0.69	1.50	0.93
TILs (10% increments)				0.89	0.80	0.98	**0.018**

HR, hazard ratio; CI, confidence interval; MRM, modified radical mastectomy.

Significant *P*‐values are shown in bold.

a For breast cancer subtype classification please see footnote to Table 2.

None of the three mRNA markers (CD3‐CD8‐FOXP3) was found to be differentially associated with DFS or OS according to paclitaxel therapy (*P*‐interaction: 0.39, 0.43 and 0.65 for DFS; 0.77, 0.66, and 0.96 for OS, respectively), suggesting lack of predictive value of the markers for paclitaxel treatment. Also, no significant interaction was observed between any of the three T‐cell markers and ER/PgR status (*P*‐interaction: 0.11, 0.87, and 0.39 for DFS; 0.43, 0.46, and 0.59 for OS, respectively). Of note, a significant interaction was observed between HER2 status and CD3 with respect to DFS (*P*‐interaction = 0.032), while a marginally significant interaction was observed between FOXP3 and HER2 status with respect to OS (*P*‐interaction *P* = 0.065) (Table S4). In the HER2‐positive and HER2‐negative subgroups of patients, the unadjusted HR (95% CI) of DFS associated with low CD3 expression was 2.08 (1.31‐3.31), *P* = 0.002 and 1.20 (0.92‐1.56), *P* = 0.19, respectively. Similarly, the unadjusted HR (95% CI) of OS associated with low FOXP3 in the HER2‐positive and HER2‐negative subgroups of patients was as follows: 1.76 (1.07‐2.88), *P* = 0.026 and 0.98 (0.72‐1.33), *P* = 0.89, respectively. In multivariate models, only the interaction between CD3 expression and HER2 status was statistically significant (adjusted *P*‐interaction = 0.037); however, the associations between any of the three T‐cell markers and DFS or OS among the HER2‐positive or HER2‐negative subgroups were of no statistical significance (Table S6). Similarly, in the triple‐negative subgroup, none of the three examined markers showed any prognostic significance with respect to DFS (Table S7).

## 4 DISCUSSION

Primary goal of our study was to evaluate the prognostic value of CD3, CD8, and FOXP3 mRNA expressions, as markers for T‐cell infiltration, in early‐stage breast cancer patients treated with anthracycline‐based adjuvant chemotherapy in the context of two prospective phase III randomized trials. Furthermore, following results of a recent work of our group, we investigated the association of RANK, RANKL, and OPG mRNA expressions with the above TILs subpopulations. Our results showed that aggressive tumors (high Ki67 protein expression) present with high CD3 mRNA expression. Likewise, high CD3, CD8, or FOXP3 mRNA expression was more frequently detected in tumors of higher histological grade and negative ER/PgR status. In the univariate analyses, high CD3 and CD8 mRNA expression was found to be of favorable prognostic value for DFS. In the multivariate analyses, the association of high CD8 mRNA expression with increased DFS was retained, whereas that of high CD3 mRNA expression was of marginal statistical significance. None of the markers was found to be of predictive value for paclitaxel treatment, despite the fact that all three markers were more highly expressed in paclitaxel‐treated patients; the lack of predictive value was probably due to the small number of patients that were not treated with paclitaxel (17% of the total population). Moreover, a significant interaction was observed between HER2 status and CD3 mRNA expression with respect to DFS. In the HER2‐positive subgroup, the hazard ratio associated with high CD3 mRNA expression was of greater magnitude compared to the hazard ratio for the entire cohort.

In the present study, CD3 and CD8 mRNA derives solely from TILs, as no staining of CD3 or CD8 has ever been reported in breast cancer cells, whether such staining was evaluated by classic IHC^48^ or the AQUA method.^49^ As for FOXP3, a very small percentage of breast cancer cells might show weak nuclear staining, which is detectable in only 1% of breast cancer cases.^50^ Therefore, most if not all of the FOXP3 mRNA expression detected in our study comes from TILs, as well and not tumor cells.

Our results demonstrate that the extent of effector lymphocyte infiltration affects survival in BC, even when we analyzed all BC variants together. Mahmoud et al^51^ found that tumor infiltration by high numbers of CD8‐positive T lymphocytes is an independent prognostic factor for improved survival in BC patients. Despite that, the clinical significance of TILs is more pronounced in more aggressive subtypes and in the absence of hormone receptors. Our findings are in accordance with those from other large clinical trials, which showed a strong association of increased lymphocyte infiltration and improved outcomes in more aggressive BC subtypes, such as TN and ER/PgR‐negative/HER2‐positive patients. A total of 2009 node‐positive breast cancer samples from the BIG 02‐98 adjuvant phase III trial were analyzed for TILs and results showed that in HER2‐positive patients, increased lymphocyte infiltration was significantly associated with benefit from anthracycline‐only chemotherapy.^52^ These findings were confirmed in an analysis using samples from the FinHER adjuvant phase III trial, where in TN patients and HER2‐positive patients receiving trastuzumab, each 10% increase in lymphocytic infiltration was significantly associated with decreased distant recurrence.^8^ Both TN and HER2‐positive patients exhibit relatively higher numbers of TILs compared to their hormone positive counterparts, with the major effect in survival benefit attributed to CD8‐positive lymphocytes. Liu et al^10^ performed IHC for CD8 staining in 3992 breast cancer samples and demonstrated that in TN patients, intratumoral or stromal infiltration by CD8‐positive lymphocytes is an independent favorable prognostic factor for BC‐specific survival (BCSS). Ali et al used 12 439 samples from breast cancer patients and quantified CD8‐positive lymphocyte infiltration by IHC. They found that the presence of intratumoral or stromal CD8‐positive lymphocytes was associated with reduced BC‐specific mortality in ER‐negative tumors (TN and HER2‐positive). However, in ER‐positive tumors, that was not the case. Nevertheless, there was an association of CD8‐positive lymphocyte infiltration with increased BC‐specific survival in ER‐positive/HER2‐positive tumors.^53^ A systematic meta‐analysis was recently performed by Mao et al including 22 964 BC patients from 22 studies and demonstrated, as in our study, that infiltrating CD8‐positive lymphocytes were correlated with better DFS and BCSS, but not OS, in the overall population. Moreover, there was an association with improved BCSS in ER‐negative, HER2‐positive, and TN subtypes, but not in the ER‐positive one.^54^

The prognostic value of FOXP3‐positive Tregs in BC is not so clear and results of several studies are controversial. Tregs may be recruited by the tumors and can suppress the antitumor activity of effector T cells in the tumor site. In our study, we showed that FOXP3‐positive Treg infiltration is not associated with DFS or OS in the overall BC population. Bates et al^15^ demonstrated that in BC samples, high rate of Treg infiltration was more frequent in patients with ER‐negative, high‐grade tumors, and positive lymph nodes and that it was associated with poor outcomes, even in ER‐positive tumors. Our finding showing that patients with ER/PgR‐negative, HER2‐positive, and grade III‐IV tumors had higher FOXP3 mRNA expression are in agreement with Bates et al^15^; however, in our study, higher FOXP3 mRNA expression was not associated with outcome. On the contrary, Liu et al^16^ found that high numbers of FOXP3‐positive cells in HER2‐positive tumors may predict chemosensitivity, whereas Lee et al^55^ demonstrated that patients with highly infiltrated TN tumors by Tregs have improved survival. Mao et al^54^ showed in a large meta‐analysis that high Treg infiltration was associated with poor OS and BCSS only in ER‐positive patients and not in ER‐negative patients. Finally, in our study, postmenopausal women were found to have higher FOXP3 mRNA expression and so did older women. The latter is in agreement with Gregg et al^56^ showing that circulating Tregs are increasing with age in healthy subjects, while it appears that the observed higher mRNA expression of FOXP3 in postmenopausal women with breast cancer is reflecting the general increase in Tregs with age.

The RANK/OPG/RANKL pathway in BC is involved in many intracellular processes, and its role as prognostic factor for BCSS or the formation of bone metastasis is unclear. We have recently shown that low RANKL mRNA expression in tumor tissue is associated with reduced DFS and bone metastases development,^19^ but other studies have demonstrated conflicting results.^57^, ^58^ In the current study, we found that there is a strong association of high RANK and RANKL mRNA expression with CD3‐positive, CD8‐positive, and FOXP3‐positive lymphocytes, while high OPG mRNA expression was associated only with FOXP3‐positive infiltrating lymphocytes. This novel finding could possibly mean that the inactivation of the RANK/RANKL pathway, resulting from increased mRNA expression of the decoy receptor OPG, might induce Treg recruitment in the tumor site or vice versa. To elucidate that, a series of preclinical and clinical studies are needed in order to make a therapeutic intervention, for example, administration of RANKL inhibitor, to abrogate Treg‐mediated immunosuppression at the tumor site.

In conclusion, our study is the first, to our knowledge, that used CD3, CD8, and FOXP3 mRNA expression as surrogate markers of T‐cell infiltration and examined the prognostic value of such markers in BC. Limitations of our study includes the relative disadvantages of using mRNA from FFPE samples due to RNA degradation and fragmentation during the fixation process and the missing information of the spatial distribution of immune cells within the tumor in comparison with the IHC method; with the latter limitation in mind, we are in the process of evaluating CD3, CD8, and FOXP3 protein expression in the same patient cohort, in order to verify whether the qRT‐PCR findings correlate to the IHC results. Nevertheless, our study clearly showed that high CD8 mRNA expression is associated with improved outcome in BC and such prognostic value is more profound in the HER2‐positive subtype. This is in agreement with other studies that used IHC for TILs evaluation. Moreover, due to the fact that we included patients from randomized trials before trastuzumab was approved as adjuvant treatment, our findings are unbiased from its use in the adjuvant setting. Further investigations of the role of TIL subtypes and their interactions with other immune cells, such as TAMs, as well as other factors and pathways, such as the RANK/OPG/RANKL pathway, could guide immunotherapeutic decisions in the future.
